# Mobility impairment and bad feet … who'd of guessed? The Foot Disease in Inpatients Study (FDIS)

**DOI:** 10.1186/1757-1146-8-S2-O26

**Published:** 2015-09-22

**Authors:** Peter A Lazzarini, Vanessa Ng, Suzanne S Kuys, Maarten C Kamp, Michael C d'Emden, Courtney Thomas, Jude Wills, Ewan M Kinnear, Scott Jen, Sheree E Hurn, Lloyd Reed

**Affiliations:** 1 School of Clinical Sciences, Queensland University of Technology, Brisbane, Queensland, 4059, Australia; 2 Allied Health Research Collaborative, Metro North Hospital & Health Service, Queensland Health, Brisbane, Queensland, 4032, Australia; 3 Department of Podiatry, Metro North Hospital & Health Service, Queensland Health, Brisbane, Queensland, 4032, Australia; 4 Musculoskeletal Research Program, Griffith Health Institute, Griffith University, Gold Coast, Queensland, 4222, Australia; 5 Department of Endocrinology and Diabetes, Royal Brisbane and Womens Hospital, Brisbane, Queensland, 4029, Australia; 6 School of Medicine, The University of Queensland, Brisbane, Queensland, 4072, Australia; 7 Department of Podiatry, North West Hospital & Health Service, Mount Isa, Queensland, 4825, Australia; 8 Department of Podiatry, Central Queensland Hospital & Health Service, Rockhampton, Queensland, 4700, Australia; 9 Department of Podiatry, West Moreton Hospital & Health Service, Queensland Health, Ipswich, Queensland, 4305, Australia

**Keywords:** Foot, inpatients, disease, complications, prevalence

## Background

Foot complications have been found to be predictors of mobility impairment and falls in community dwelling elderly patients. However, fewer studies have investigated the link between foot complications and mobility impairment in hospital in patient populations. The aim of this paper was to investigate the associations between mobility impairment and various foot complications in general inpatient populations.

## Methods

Eligible participants were all adults admitted overnight, for any reason, into five diverse hospitals on one day; excluding maternity, mental health and cognitively impaired patients. Participants underwent a foot examination to clinically diagnose different foot complications; including foot wounds, infections, deformity, peripheral arterial disease and peripheral neuropathy. They were also surveyed on social determinant, medical history, self-care, footwear, foot complication history risk factors, and, mobility impairment defined as requiring a mobility aid for mobilisation prior to hospitalisation.

## Results

Overall, 733 participants consented; mean(±SD) age 62(±19) years, 408 (55.8%) male, 172 (23.5%) diabetes. Mobility impairment was present in 242 (33.2%) participants; diabetes populations reported more mobility impairment than non-diabetes populations (40.7% vs 30.9%, p < 0.05). In a backwards stepwise multivariate analysis, and controlling for other risk factors, those people with mobility impairment were independently associated with increasing years of age (OR = 1.04 (95% CI) (1.02-1.05)), male gender (OR = 1.7 (1.2-2.5)), being born in Australia (OR = 1.7 (1.1-2.8), vision impairment (2.0 (1.2-3.1)), peripheral neuropathy (OR = 3.1 (2.0-4.6) and foot deformity (OR = 2.0 (1.3-3.0).

## Conclusions

These findings support the results of other large studies investigating community dwelling elderly patients that peripheral neuropathy and foot deformity are independently associated with mobility impairment and potentially falls. Furthermore the findings suggest routine clinical diagnosis of foot complications as defined by national diabetic foot guidelines were sufficient to determine these associated foot complication risk factors for mobility impairment. Further research is required to establish if these foot complication risk factors for mobility impairment are predictors of actual falls in the inpatient environment.

